# Screening differential circular RNA expression profiles reveals the regulatory role of circTCF25-miR-103a-3p/miR-107-CDK6 pathway in bladder carcinoma

**DOI:** 10.1038/srep30919

**Published:** 2016-08-03

**Authors:** Zhenyu Zhong, Mengxin Lv, Junxia Chen

**Affiliations:** 1 The First Clinical College, Chongqing Medical University, Chongqing 400016, China; 2 Department of Cell Biology and Genetics, Chongqing Medical University, Chongqing 400016, China

## Abstract

Circular RNAs (circRNAs), a kind of non-coding RNAs, have shown large capabilities in gene regulation. However, the mechanisms underlying circRNAs remain largely unknown so far. Recent studies demonstrated that circRNAs play miRNA sponge effects and regulate gene expression by microRNA response elements. Here, we screened circRNA expression profiles of bladder carcinoma using microarray assay. A total of 469 dysregulated circular transcripts are found in bladder cancer compared with normal tissues, among which 285 were up-regulated and 184 were down-regulated. Six circRNAs were identified to have significant differences by qRT-PCR. We speculated that circRNAs might involve in cancer-related pathways via interactions with miRNA by multiple bioinformatical approaches. Therefore, we further predicted that circTCF25 could sequester miR-103a-3p/miR-107, which potentially lead to the up-regulation of thirteen targets related to cell proliferation, migration and invasion. Subsequently, we demonstrated that over-expression of circTCF25 could down-regulate miR-103a-3p and miR-107, increase CDK6 expression, and promote proliferation and migration *in vitro* and *vivo*. This is the first study to exploit circRNA profiling and circRNA/miRNA interactions in bladder cancer. Our work laid the foundation to investigate the functions of circRNAs in cancers. The data also suggest that circTCF25 might be a new promising marker for bladder cancer.

Bladder cancer is the prevalent malignant tumor at the urinary system. The prevalence of 5-year is estimated at 1,110,265 worldwide, ranking the 9th in cancer incidence around the world and the 13th main causes of death by cancer^1^,^2^. Most clinical trials of chemotherapy for advanced bladder carcinoma displayed confined benefits^3^. Therefore, it is necessary to identify the potential molecular targets and novel pathways underlying tumorigenesis and development of bladder cancer.

Recent systematic genomic studies have revealed a broad spectrum of non-coding RNAs (ncRNAs) that are implicated in various diseases (cancers). The change of transcript patterns is not only abnormal in levels of protein-coding RNAs, but also related to the dysregulated expression of non-coding transcripts in human genome^4^. The competitive endogenous RNAs (ceRNAs) include pseudogene transcripts, long noncoding RNAs, cirular RNAs and mRNAs, and these transcripts can compete for the pool of same microRNA response elements (MREs) to influence the activities of microRNAs (miRNAs) in regulating gene expressions^5^. Theoretically, any RNA transcript with MREs might act as ceRNA. If the balance of ceRNA intricate network is disturbed, it could lead to cancer. Some studies have already shown that ceRNAs implicate in certain cancers including leukemia, Hodgkin’s lymphoma, esophageal cancer, hepatocellular carcinoma etc^6^,^7^,^8^,^9^.

The circular RNA (circRNA), as a widespread and diverse endogenous non-coding RNA with huge regulatory potency, is coming to be a novel focus in RNA field^10^. Unlike linear RNAs, circRNAs form covalently closed loop structures with neither 5′ caps nor 3′ tails tails^11^, which results in a higher stability than liner transcript and are not affected by RNase R and RNA exonuclease^12^. Furthermore, the expression of circRNAs manifests tissue and developmental stage-specificity and a fraction of circRNAs displays conservation across species^13^,^14^.

Importantly, a general phenomenon was discovered that circRNAs could be targeted by endogenous miRNAs, suppressing miRNA activity as miRNA sponge^15^. Also, the statistical measure and gene ontology (GO) enrichment analysis revealed that the protein-coding genes are associated with the miRNA/circRNA interactome^16^. Recently, it has been reported that circRNAs are correlated with cancer-associated miRNAs and the circRNA/miRNA axes are implicated in cancer-related pathways. Some artificial circRNAs have displayed significant anti-cancer effects^17^.

Thus, this emerging family of RNAs could be considered as ideal biomarkers for diagnostic and prognostic purpose and even as potential therapeutic targets in the future. However, the mechanisms of circRNAs underlying cancer development and progression remain largely unknown. Until now, no research has been reported on circRNAs in human bladder cancer.

The molecular mechanisms underlying the circRNAs in bladder cancer remain largely unknown. Therefore, we hypothesize that dysregulating circRNAs might be involved in tumorigenesis and metastasis of bladder cancer. Here, we investigated differentially expressed circRNA profile in 4 pairs of snap-frozen bladder carcinoma and para-carcinoma tissue using high-throughput microarray. The significant differential expressions of representative circRNAs were further confirmed with qRT-RCR in 40 pairs of samples. Next, we constructed a circRNA/miRNA network by bioinformatics approaches. It was found that circTCF25 could interact with miR-103a-3p and miR-107. DIANA-miRPath enrichment analysis was performed to identify molecular pathways potentially altered by the expression of those miRNAs. Then, we depicted a network containing thirteen pivotal genes correlated with circTCF25-miR-103a-3p/miR-107 axes. DAVID functional annotation was executed for these genes. Finally, we explored the functions of circTCF25 in bladder cancer in *vivo* and *vitro*. In summary, our data provided a novel basis for functional research of circRNAs in bladder cancer. The circTCF25 might be a new potential target for bladder cancer diagnosis and therapy. The study may be of biological and clinical importance.

## Results

### Identification of differentially expressed circRNA profiles

**Figure 1:**
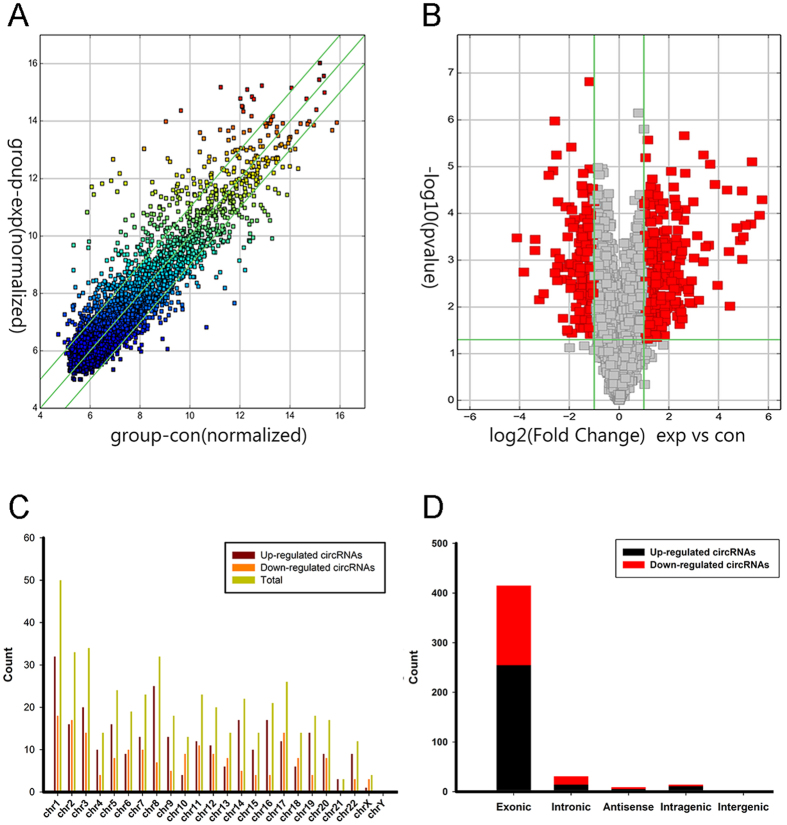
Differences and characterizations in circRNA expression profiles between bladder carcinoma tissues and adjacent non-carcinoma tissues. (**A**) Scatter plots are used to evaluate the difference in the expression of circRNAs between experiment group and control group. The values plotted on X and Y axes are the averaged normalized signal values of each group (log2 scaled). The middle green line refers to no difference between the two groups. And the flank green lines represent 2.0 fold changes. The circRNAs above the top green line and below the bottom green line indicate more than 2.0 fold changes between two groups. (**B**) Volcano plots are using for visualizing differential expression between two different conditions. The vertical lines correspond to 2.0 fold (log2 scaled) up and down, respectively, and the horizontal line represents a P-value of 0.05 (−log10 scaled). The red points in plot represent the differentially expressed circRNAs with statistical significance. (**C**) The distribution of differentially expressed circRNAs in human chromosomes. (**D**) The bar diagram shows the circRNA category. Most of differentially expressed circRNAs originate from the exons. Some are from introns, while, a few are other sources.

**Figure 2:**
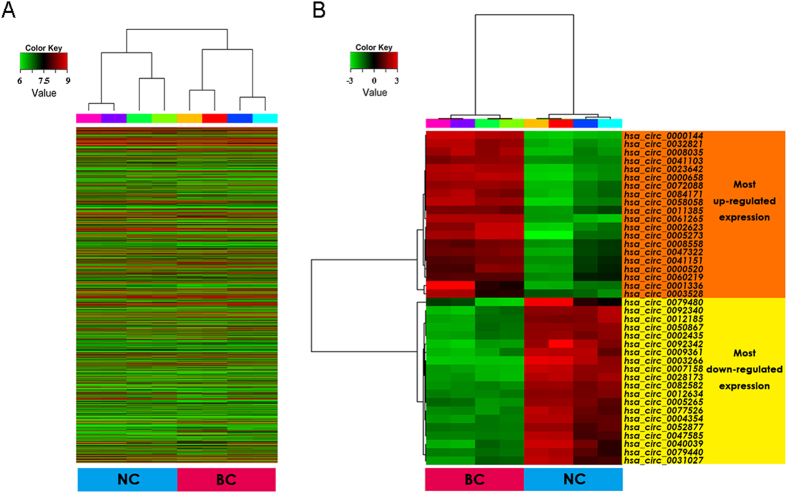
Heat map and hierarchical clustering showing expression values of all target circRNAs and the most up and down regulated circRNAs. Each column represents a sample and each row represents a circRNA. Red strip represents high relative expression and green strip represents low relative expression. BC represents bladder cancer group, and NC represents normal control group. Each group contains four different samples. (**A**) Hierarchical cluster analysis of all target circRNAs. All target circRNAs mean entities where at least 4 out of 8 samples have flags in Present or Marginal. Flags are attributes that denote the quality of the entities using methods from GeneSpring software. (**B**) Hierarchical cluster analysis of the most up and down regulated circRNAs.

High throughput microarray assay facilitated our research on circRNA expression signatures in bladder cancer. A total of 3243 circRNA targets were detected by microarray probes in four pairs of samples. Differentially expressed circRNAs were displayed through fold change filtering (Fig. 1A). And the volcano plot filtering identified differentially changed circRNAs with statistical significance between two groups (Fig. 1B). As a result, 469 circRNAs were detected to be differentially regulated by fold change ≥2.0, P < 0.05 and FDR < 0.05, among which 285 circRNAs were up-regulated while 184 circRNAs were down-regulated. Among these circRNAs, up-regulated circRNAs are more common than down-regulated circRNAs. The distribution of the circRNAs on the human chromosomes is depicted (Fig. 1C). Most of differentially expressed circRNAs are transcribed from the protein coding exons. Some are from introns. While, there are a few other sources of circRNAs (Fig. 1D). In present study, the top twenty up- and down-regulated circRNAs are listed in Table 1 by fold change. Hierarchical clustering showed that circRNA expression pattern were distinguishable between cancer and normal control samples (Fig. 2). The data suggested that the expression of circRNAs in bladder cancer tissues is different from that in matched non-tumor tissues.

### Construction of the circRNA/miRNA interaction network

**Figure 3:**
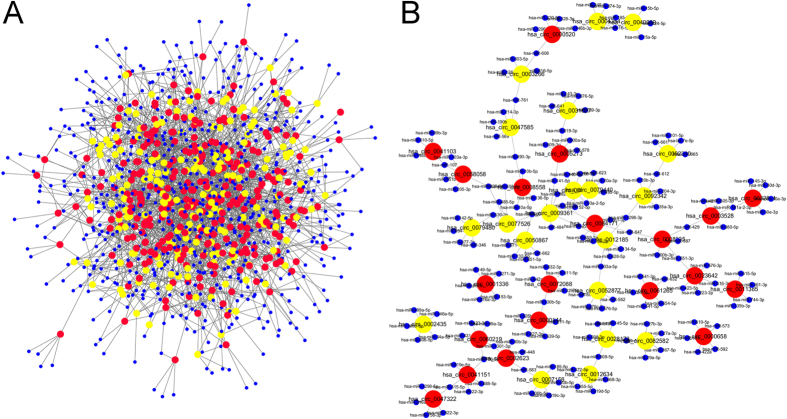
The circRNA/miRNA network analysis. (**A**) The panorama network consists of 285 up-regulated circRNAs (red nodes), 184 down-regulated circRNAs (yellow nodes) and their target miRNAs (blue nodes). They were connected by 2329 edges based on seed sequence pairing interactions. (**B**) A magnified network comprising the top 20 up-regulated and down-regulated circRNAs and their target miRNAs is presented.

To determine the function of circRNAs, interactions between circRNAs and their target miRNAs were theoretically predicted by conserved seed-matching sequence using the software for miRNA target prediction made in Arraystar according to TargetScan and miRanda database. All the differentially expressed circRNAs were predicted according to the complementary miRNA matching sequence. A total of 643 miRNAs could be combined with circRNAs. The data displayed each circRNA and its potential complementary binding miRNAs (see Supplementary Table S1). An entire network of circRNA/miRNA interaction was delineated using Cytoscape (Fig. 3A). Further, the part of the graph is enlarged to display the top 20 up- and down-regulated circRNAs and their acting miRNAs (Fig. 3B).

### Validation for the differential expression level of circRNAs

**Figure 4:**
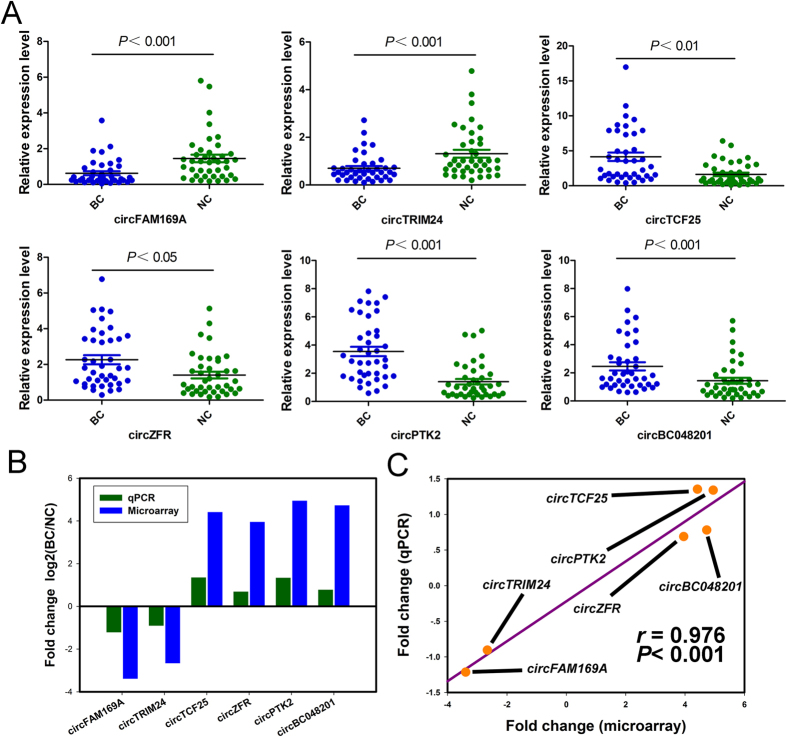
Quantitative real-time PCR validation for the expression of six circRNAs. (**A**) The expression levels of six circRNAs were validated by qPCR in forty patients. BC, bladder cancer tissues. NC, normal control. Data are shown as mean ± SEM. (**B**) The comparison between microarray data and qPCR results. The vertical axis shows the mean of fold change (log2 transformed) of each circRNAs measured by qPCR and microarray respectively. (**C**) The qRT-PCR results matched well with the microarray data. Pairwise scatter plots are showed, comparing the fold changes (log2 transformed) of the microarray (horizontal axis) and the qPCR (vertical axis). The *r* stands for Pearson’s correlation coefficient (linear correlation coefficient).

Six circRNAs were validated out using qRT-PCR in 40 pairs of samples including the tissues for microarray analysis. The results showed that these circRNAs were significantly differentially expressed. Particularly, circFAM169A (hsa_circ_0007158) and circTRIM24 (hsa_circ_0082582) are significantly down-regulated in bladder carcinoma groups, while circTCF25 (hsa_circ_0041103), circZFR (hsa_circ_0072088), circPTK2 (hsa_circ_0005273) and circBC048201 (hsa_circ_0061265) are significantly higher expressed in carcinoma tissues (Fig. 4A). The log2 fold-changes were calculated for microarray data and qPCR results (Fig. 4B). The data indicated that microarray analysis was well consistent with qPCR results regarding the expression levels of the six circRNAs (Fig. 4C).

### Prediction of circRNA-miRNA-mRNA associations

**Figure 5:**
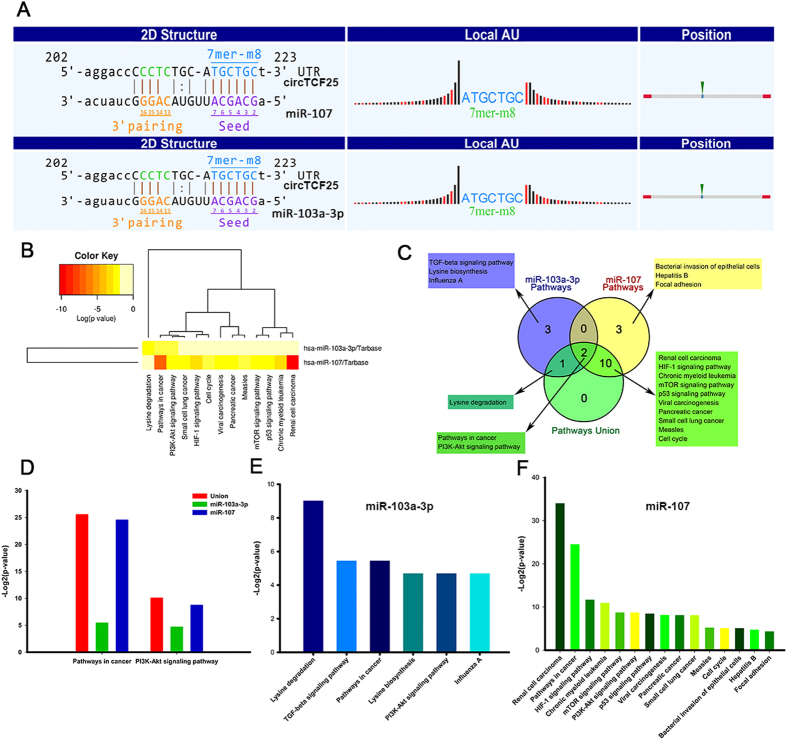
Prediction for circRNA/miRNA interactions and miRNA-mediated signaling pathways. (**A**) The interaction of circTCF25-miR-103a-3p/miR-107 was predicted based on TargetScan and miRanda. (**B**) The heatmap reveals statistically significant correlations between miR-103a-3p or miR-107 and their mediated pathways by P-value (log scaled). Red represents high significance. (**C**) The Venn diagram indicates the relevant pathways of miR-107 mediated, miR-103a-3p mediated, and miR-103a-3p and miR-107 jointly mediated. *Pathways in cancer* and *PI3K-Akt signaling pathway* are intersected. (**D**) The bar graph indicates the magnitudes of the significant correlation about *Pathways in cancer* or *PI3K-Akt signaling* among miR-107 mediated, miR-103a-3p mediated, and miR-103a-3p and miR-107 jointly mediated by P-value (−log2 scaled). (**E**,**F**) The bar graphs show the magnitudes of the significant correlations about miR-103a-3p and miR-107 mediated signaling pathways respectively by P-value (−log2 scaled).

In order to explore the molecular mechanism of circRNAs, the circRNA-miRNA-mRNA axis in cancer-related pathways was predicted. DIANA-miRPath determined all the candidate miRNAs that are involved in possible pathways by p-value cutoff at 0.05. The data showed that circTCF25 was implicated in cancer-related pathways for bladder cancer with the greatest potential. Firstly, it was predicted that circTCF25 could harbor miR-103a-3p and miR-107 by miRNAs seed sequence matching (Fig. 5A). Then, DIANA-miRPath analysis revealed that both miR-103a-3p and miR-107 were associated with cancer-related pathways (Fig. 5B). Moreover, DIANA-miRPath demonstrated that a total of six miR-103a-3p associated pathways, fifteen miR-107 associated pathways and thirteen merged miR-103a-3p and miR-107 related pathways. The venn diagram revealed the intersection (Fig. 5C). The data showed that the *pathways in cancer and PI3K-Akt signaling pathway* are the crossing pathways related to miR-103a-3p and miR-107. In addition, the statistically significant correlations (p-value) between the two pathways was compared. We found that *pathways in cancer* were much more significant than *PI3K-Akt signaling pathway* (Fig. 5D). Similarly, *Pathways in cancer* were also the most significant pathways for miR-103a-3p (Fig. 5E) and miR-107 (Fig. 5F) mediated pathways respectively. These findings suggest that circTCF25-miR-103a-3p/miR-107 axes might participate in *Pathways in cancer*.

### Annotation for target genes of circTCF25-miR-103a-3p/miR-107 axes

**Figure 6:**
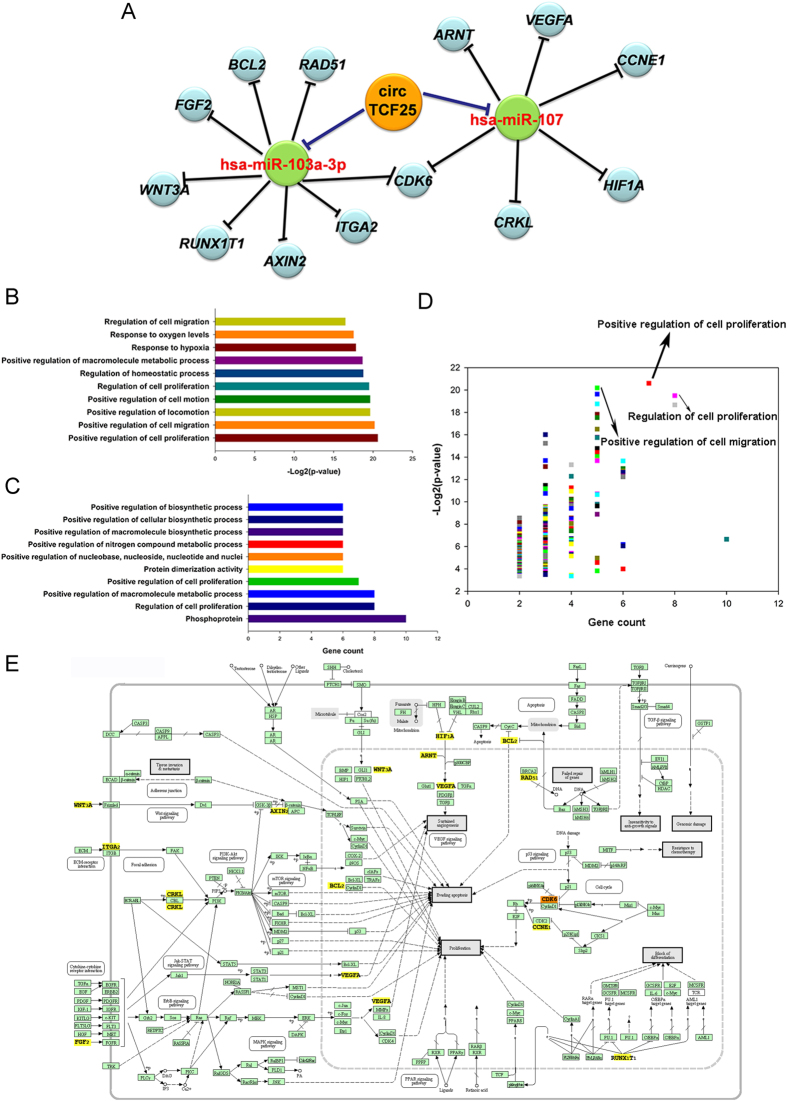
Function annotations for target genes mediated by circTCF25-miR-103a-3p/miR-107 axes. (**A**) The bioinformatical prediction of circTCF25 target genes related to *Pathways in cancer*. The total of 16 nodes and 16 edges are constructed based on base-pairing interactions. (**B**,**C**) DAVID function annotation for the miR-103a-3p and miR-107 targeted genes of *Pathways in cancer*. The vertical axis shows the annotated functions of the target genes. The horizontal axes shows −log2 transformed P-value and the gene number of each cluster respectively. Only the most significantly enriched clusters are included. Details are in Supplementary Table S2. (**D**) The scatter plots demonstrate all cluster features about P-value and gene count, and the plots on the top right corner represent high significance and more genes. Positive regulation of cell proliferation and migration are labeled with high significance and more genes. (**E**) Mapping of *Pathways in cancer* mediated by miR-103a-3p and miR-107. In this map, circRNAs could act as positive regulators of target genes, while miRNAs function as negative regulators. These yellow target genes could be regulated by circTCF25-miR-103a-3p/miR-107 axes to devote to the initiation and progression of bladder cancer.

To investigate the functional effects of circTCF25-miR-103a-3p/miR-107 axes, we predicted target genes of miR-103a-3p/miR-107 associated with *Pathways in cancer* using DIANA-miRPath. The data showed that a total of thirteen genes could be regulated by circTCF25-miR-103a-3p/miR-107 axes. As a result, a network of circRNA/miRNA/mRNA interactions was established (Fig. 6A). Then, the DAVID functional annotation was performed for target genes. The analysis revealed that these genes were significantly correlated with cell proliferation and migration (Fig. 6B–D and Supplementary Table S2). The data were integrated from KEGG and DIANA-miRPath to draw the cancer-related signaling network containing thirteen genes on circTCF25-miR-103a-3p/miR-107 axes (Fig. 6E). In the network, it has been known that CDK6 and CCNE_1_ could act on the process of cell cycle and cell proliferation. BCL2 functions as a suppressor of apoptosis. Moreover, RAD_51_ could impede the repair of genes, and RUNX_1_T_1_ might block the differentiation of cancer cells. HIF_1_A, ARNT and VEGFA are involved in VEGF signaling pathway to promote angiogenesis. In addition, CRKL could activate PKB/Akt/mTOR pathway, WNT_3_A could stimulate Wnt/β-catenin signaling pathway and FGF_2_ might trigger ErbB signaling pathway. All of those are considered to be closely related to angiogenesis, tumorigenesis and metastasis.

### Determination for the regulatory role of circTCF25-miR-103a-3p/miR-107-CDK6 axes

**Figure 7:**
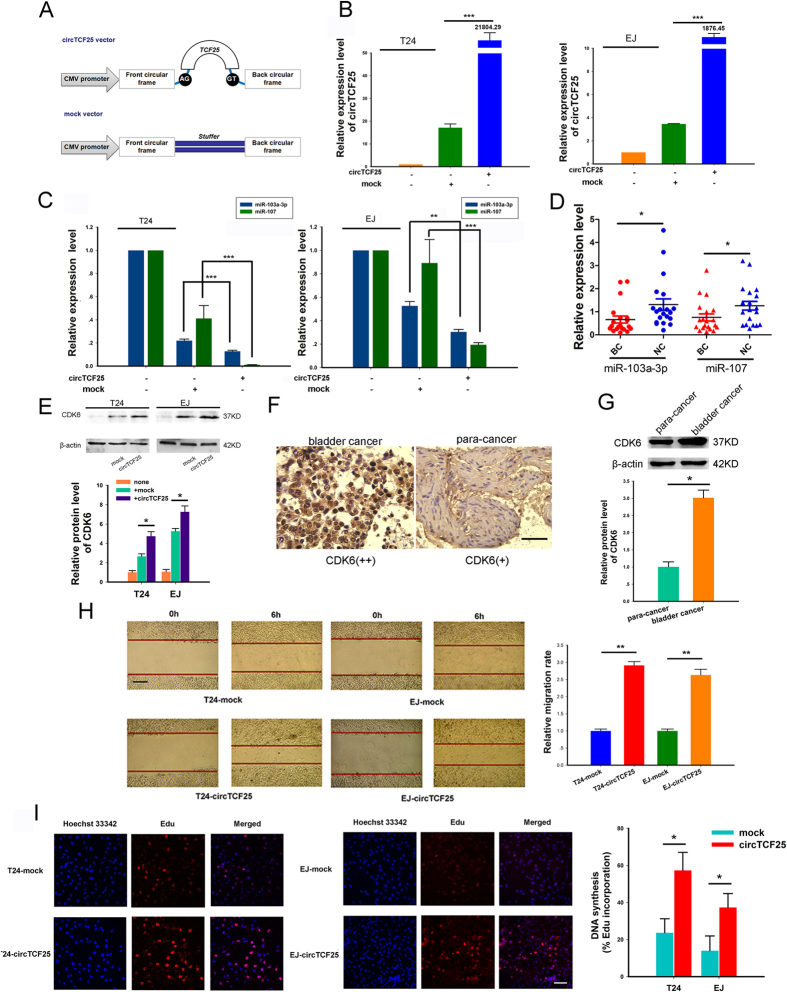
The circTCF25 promotes proliferation and migration of bladder cancer cells via circTCF25-miR-103a-3p/miR-107-CDK6 pathway. (**A**) The sketch of structure of circTCF25 and mock vector are shown. Both of them consist of cytomegalovirus promoter, front circular frame (flanking genomic sequence) and back circular frame (inverted upstream sequence). But only circTCF25 vector includes splice sites and TCF25 encoding sequence. (**B**) Real-time qPCR analysis shows the expression levels of circTCF25 after transfection in T24 and EJ cells. Over-expression of circTCF25 has been confirmed compared to control. Data are shown as mean ± SD, ***P < 0.001, n = 3. (**C**) Over-expression of circTCF25 down-regulates the expression of miR-103a-3p and miR-107 significantly compared with mock vector group in T24 and EJ cells by qPCR. Mean ± SD, **P < 0.01, ***P < 0.001, n = 3. (**D**) The expression levels of miR-103a-3p and miR-107 in twenty-pair samples. BC, bladder carcinomas. NC, adjacent normal controls. Mean ± SEM. *P < 0.05. (**E**) Western blot analysis shows the CDK6 protein level in EJ and T24 cells after transfection with circTCF25, mock and nothing. β-actin is internal control. *P < 0.05, n = 3. (**F**) Representative photographs show immunohistochemical staining of tissues in (**D**) with antibody of CDK6. Scale bar, 100 μm. Bladder tumor tissues showed stronger brown staining for CDK6 compared with para-cancer tissues. (**G**) Western blot analysis of CDK6 using tissues in (**F**), *P < 0.05. (**H**) Would healing assay evaluates cell migration capability. The width of wound is measured in three-independent wound sites of each group at 0 and 6 hour. Scale bar, 200 μm. Mean ± SD, **P < 0.01. (**I**) Edu proliferation assay determines cell proliferation ability. More red granules are observed in T24-circTCF25 and EJ-circTCF25 cells, reflecting more active DNA replication. The nuclei are stained by Hoechst 33342. Scale bar, 100 μm. Mean ± SD, *P < 0.05.

CDK6 could be targeted by both miR-103a-3p and miR-107 in the network, suggesting that it might be a crucial factor mediated by circTCF25-miR-103a-3p/miR-107 axes. Therefore, we supposed that circTCF25 could play a positive regulatory role on CDK6 to stimulate cell proliferation in bladder cancer. The circTCF25 vector was constructed that could produce circTCF25 transcripts. Meanwhile, a mock empty vector has also been constructed to serve as a control (Fig. 7A). After transfection, over-expression of circTCF25 was identified using qPCR in T24 and EJ cells (Fig. 7B). Then, we observed that the expressions of miR-103a-3p and miR-107 were down-regulated significantly in over-expression circTCF25 group compared with mock control in T24 and EJ cells (Fig. 7C). Meanwhile, the expression levels of miR-103a-3p and miR-107 in bladder carcinoma tissues were lower than those in para-carcinoma tissues by qPCR (Fig. 7D). Western blot analysis showed that up-regulating circTCF25 could increase the protein level of CDK6 both in EJ and T24 cells (Fig. 7E). Higher expression of CDK6 has been consistently detected in human bladder carcinoma tissues with immunohistochemistry and Western blot (Fig. 7F,G). Moreover, T24-circTCF25 and EJ-circTCF25 cells exhibited faster proliferation and stronger migration by Edu and wound healing assays compared with control respectively (Fig. 7H,I). More red spots were observed in T24-circTCF25 and EJ-circTCF25 cells, indicating an active DNA replication and a vigorous growth in Edu assay. These results suggest that circTCF25 could promote the proliferation, migration, tumor growth and metastasis through circTCF25-miR-103a-3p/miR-107-CDK6 pathway in bladder cancer.

## Discussion

In the past years, the existence of circRNAs was occasionally identified with covalent linkages in animal cells. The circRNA was formerly considered to be rare in nature, or even disregarded as noise and artifact of transcription^18^,^19^,^20^. However, with high-throughput sequencing and bioinformatic analysis, circRNAs are recently identified to be abundant stable ncRNAs^14^,^21^. However, in contrast to the plentiful knowledge of biogenesis, to date, the function of circRNAs has been rarely studied. Fortunately, the advent and utilization of circRNA microarray facilitated our research on screening differentially expressed circRNAs and exploring their functions.

In the present study, we first reported on circRNA profiles in bladder cancer. Our circRNA expression profiles revealed that 285 circRNAs are aberrantly up-regulated and 184 circRNAs are down-regulated in bladder carcinoma tissues compared with their paired adjacent noncancerous tissues. In the expression signatures, more than twenty circRNAs are up or down regulated with fold change > 10, which suggests that these significantly differentially expressed circRNAs might be implicated in oncogenesis and development of bladder cancer. Especially, circFAM169A, circTRIM24, circTCF25, circZFR, circPTK2 and circBC048201 were confirmed to be significantly dysregulated in bladder carcinoma compared with adjacent non-tumor tissues by qRT-PCR. One recent study reported that 400 circRNAs were present in human cell free saliva^22^. It has been showed that hundreds of circRNAs have higher expressions than homologous linear transcripts in blood. The circRNA isoforms are easily detected but the corresponding linear mRNAs are actually absent. Therefore, circRNA could embody disease information that cannot be determined by conventional RNA assay, suggesting that the circRNAs are promising biomarkers for clinical application in the future^23^. Therefore, our findings indicated that circFAM169A, circTRIM24, circTCF25, circZFR, circPTK2 and circBC048201 represent potentially valuable diagnostic biomarkers for bladder cancer. The six circRNAs are being further tested in our laboratory with tissues, plasma, and urine.

In addition, our study, for the first time, predicted hundreds of circRNA/miRNA interactions based on conserved seed sequence matches. An interaction network of circRNA/miRNA was constructed, which provided valuable information for ceRNA research of circRNAs. The discovery of circRNAs expanded competing endogenous RNAs members, for the complementarity of circRNAs to their linear mRNA counterparts^24^. The model of ceRNAs interplay was clarified in Supplementary Figure S1, where miRNAs, mRNAs and circRNAs could keep dynamic equilibrium of cellular homeostasis. It is proposed that when the abundance of miRNAs vastly exceeds that of the ceRNAs, most targeted mRNAs would be completely suppressed. On the contrary, when the number of ceRNAs surpass their miRNAs, the competition would be minimizing because of the limited miRNAs available^5^,^25^. Recently, it has been known that circRNAs could play miRNA sponge effects and might compete against other endogenous RNAs^14^,^15^,^26^. Those circRNAs harboring miRNA binding sites could be potential candidates of ceRNAs. For instance, the circRNA *CDR1as* contains conserved seed matches to miR-7 and the expression levels of miR-7 targets were decreased when silencing of *CDR1as*, while, over-expression of *CDR1as* prevented the downregulation of those targets^14^,^15^,^27^. It has been demonstrated that miR-7 involves in numerous pathways, for miR-7 could directly down-regulates a number of oncogenes including EGFR, Raf1, Pak1, Ack1, IGF1R, PIK3CD and mTOR^17^. Lately, the report showed that the expression of circRNA *ITCH* was usually low in esophageal squamous cell carcinoma compared with normal tissues. The cir-ITCH could increase the ITCH production, decoying miR-7, miR-17, and miR-214. Up-regulating ITCH accelerates ubiquitination and degradation of phosphorylated Dvl2, and thereby restrains the Wnt/β-catenin pathway^28^. Theoretically, any RNA transcripts with MREs could sequester miRNAs and act as ceRNA. If the intricate ceRNA network is perturbed, it may lead to cancer. The illumination of RNA interaction would supply new discernment for the underlying mechanisms of oncogenesis and lay the basis for searching new therapy against cancers. It is not clear whether all circRNAs function as miRNA sponges of miRNA. To date, there has been no report on ceRNAs in bladder cancer.

Our results showed that the circTCF25 was highly expressed in bladder cancer compared to control. With bioinformatical methods, we found that circTCF25 have MREs of miR-103a-3p and miR-107. Therefore, we speculated that circTCF25 might competitively bind with miR-103a-3p and miR-107 and relieve inhibitory effects on associated target genes. DIANA-miRPath analysis showed that circTCF25 may be implicated in cancer-associated pathways through targeting miR-103a-3p and miR-107. Integrating diverse bioinformatical data, we depicted a circRNA-miRNA-mRNA regulatory pathway in bladder cancer. The bioinformatical results infer that the circTCF25 could sequester miR-103a-3p and miR-107, which trigger up-regulating expressions of RAD51, BCL2, CDK6, RUNX1T1, AXIN2, ITGA2, FGF2, WNT3A, CRKL, ARNT, CDK6, HIF1A, CCNE1 and VEGFA in the network (Fig. 6A). DAVID functional annotations for those target genes revealed that the circTCF25-miR-103a-3p/miR-107 axes are implicated in positively regulating cell proliferation and migration. In order to get a general view, a network diagram of crosstalk among these pathways was constructed based on circTCF25-miR-103a-3p/miR-107 axes (Fig. 6E). The data showed that circTCF25-miR-103a-3p/miR-107 axes could play regulatory roles in increasing proliferation, evading apoptosis, blocking differentiation and promoting angiogenesis. These results suggested that circTCF25 could act as a regulator in oncogenesis and metastasis of bladder cancer. Li *et al*. proposed that circRNAs could correlate with cancer miRNAs and the circRNA/miRNA axes might participate in cancer-related pathways^17^, which is consistent with our analysis results.

In order to further explore the functions of circTCF25-miR-103a-3p/miR-107 axes, over-expression vectors of circTCF25 were transfected into bladder cancer EJ and T24 cells. The results showed that up-regulating circTCF25 could inhibit miR-103a-3p and miR-107 expressions, increase CDK6 protein level, and promote proliferation and migration of EJ and T24 cells. Compared to the adjacent tissues, we found that the circTCF25 and CDK6 were highly expressed in bladder carcinoma tissues, while, miR-103a-3p and miR-107 were lowly expressed in bladder cancer. Previous studies showed that CDK6 along with Cyclin D steers cell cycle process from G_1_ to S phase via the phosphorylation and inactivation of the RB1^29^. Also, it has been reported that miR-103a-3p and miR-107 could negatively regulate CDK6 and other oncogenic factors^30^,^31^. Our experiment results agreed with these findings. According to bioinformatical prediction and our experiment data, the circTCF25, an emerging circular RNA molecule, might play an important regulatory role via the miR-103a-3p and miR-107 in bladder cancer development.

In conclusion, for the first time, our study revealed the circRNA expression signatures of bladder carcinoma. We established a novel approach for comprehensively screening circRNAs that could be involved in cancer-related pathways with different bioinformatical analysis. Furthermore, the regulatory role of circTCF25-miR-103a-3p/miR-107-CDK6 pathway was preliminarily confirmed in bladder cancer. Next, the further research on functions and mechanisms underlying circTCF25 is being carried out in our laboratory. Our findings highlighted the possibility that circTCF25 could serve as a valuable target for bladder cancer. These data laid a foundation for further diagnostic, therapeutic and functional research of circRNAs in bladder carcinoma.

## Methods

### Ethics statement

This study was approved and supervised by The Ethics Committee of Chongqing Medical University. All the experiments were carried out in accordance with UK Home Office regulations. Human bladder carcinoma tissues and surrounding tissues were obtained from the patient receiving operation in The First Affiliated Hospital of Chongqing Medical University. Written informed consents were obtained from patients for research purposes.

### Clinical specimens

Four pairs of snap-frozen bladder carcinoma tissue and matched para-carcinoma tissue were recruited from the Hospital Clinic for circRNA microarray analysis. Subsequently, a total of forty-pair samples including the samples for microarray analysis were used for the circRNA validation by reverse transcriptase quantitative (RT-q) PCR. All the experimental subjects were consecutive patients and did not receive any other treatment prior to operation. All the bladder carcinomas were confirmed by experienced pathologists. Clinical and pathologic characteristics of patients were determined according to WHO/ISUP classification and UICC TNM classification and are presented in Supplementary Table S3.

### RNA extraction

Total RNA was isolated from frozen tissues in liquid nitrogen using TRIzol (Invitrogen, Carlsbad, CA) according to instructions. RNA quantity and quality were evaluated by Nano Drop ND-1000 spectrophotometer (Nano Drop Thermo, Wilmington, DE) and RNA integrity was assessed by agarose gel electrophoresis.

### Microarray hybridization

Isolated total RNA was treated with Rnase R (Epicentre, Inc.) to remove linear RNA, and then was amplified and transcribed into fluorescent cRNA using random primer according to Arraystar Super RNA Labeling protocol. Next, the labeled cRNA was purified by RNeasy Mini Kit (Qiagen, Germany). Specific activity (pmol Dye/μg cRNA) and concentration (μg/μl) of cRNA were surveyed to assess the labeling efficiency by Nano Drop ND-1000. After cRNA preparation, 1 μg labeled cRNA, mixed with 1 μl 25× Fragmentation Buffer and 5 μl 10× Blocking Agent, was heated at 60 °C for half an hour. After deliquating the labeled cRNA with 25 μl 2× Hybridization buffer, 50 μl hybridization solution was added into the gasket slide and assembled to circRNA expression microarray. The slide was incubated at 65 °C for seventeen hours in Agilent Hybridization Oven. After washing, slides were fixed and scanned to generate images using Agilent Scanner G2505C. And acquired array images were analyzed by Agilent Feature Extraction software. The microarray hybridization and the collection of data were performed by KangChen Bio-tech, Shanghai, China.

### Microarray data analysis

Quantile normalization and subsequent data processing were executed using R software package. CircRNAs with at least 4 out of 8 samples that have flags (an attribute that denotes the quality of the entities) in Present or Marginal were considered to be target according to GeneSpring software’s definitions and instructions. And target circRNAs were retained for further differential analysis. Comparing two groups (bladder cancer versus normal control) of profile differences, the fold change (i.e. the ratio of the group averages) for each circRNA was computed. Paired t test was performed to analyze the statistical difference. The false discovery rate (FDR) is applied to determine the threshold of P-value. An FDR < 0.05 was recommended. CircRNAs (fold changes ≥ 2.0 and P-values < 0.05) were differentially expressed with statistical significance.

### Hierarchical clustering analysis

To generate an overview of circRNA expression profiles between the two groups, the hierarchical clustering analysis was performed based on expression value of all target circRNAs and the most significant differentially expressed circRNAs using Cluster and TreeView program.

### Delineation of circRNA/miRNA interactions

The circRNA/microRNA interaction was predicted using Arraystar’s home-made miRNA target prediction software based on miRanda and TargetScan^32^,^33^. To establish circRNA-miRNA network, we searched MREs on circRNAs using the software, then selected the miRNAs according to seed match sequences. The graph of the circRNA/miRNA network was drawn with the help of Cytoscape 3.01.

### Quantitative real-time PCR

After RNA extraction, M-MLV reverse transcriptase (Invitrogen, Carlsbad, CA) was used for synthesizing cDNA according to manufacturer’s instructions. The expression level of the circRNAs was evaluated by qPCR using SYBR Green assay. Specific divergent primers were designed to amplify the circular transcripts. PCR was performed in 10 μl reaction volume, including 2 μl of cDNA, 5 μl 2× Master Mix, 0.5 μl of Forward Primer (10 μM), 0.5 μl of Reverse Primer (10 μM) and 2 μl of double distilled water. The reaction was set at 95 °C for 10 min for pre-denaturation, then at 95 °C for 10 s and at 60 °C for 60 s repeating 40 cycles. β-actin was used as a reference. Both target and reference were amplified in triplicate wells. And the relative level of each circRNA was calculated using 2^−△△Ct^ method.

### Prediction for circRNA-miRNA-mRNA pathways

The miRNA pathway investigating was carried out based on DIANA-miRPath^34^. P < 0.05 was used as the criterion for statistical significance. The miRNA target genes implicated in pathway were introduced into wiring diagrams of interaction networks among genes by the KEGG database^35^. Also, the function of those target genes were predicted and annotated in the network using Database for Annotation, Visualization and Integrated Discovery (DAVID)^36^. In brief, the circRNA-miRNA pathway optimization is carried out as following steps (see Supplementary Figure S2). Firstly, we searched miRNA candidates associated cancer-related pathways in the circRNA/miRNA interaction network. Subsequently, functional annotations were performed for target genes in cancer-related pathways. Finally, elaborated signaling pathway networks were constructed gathering target genes and interacting proteins.

### Vector construction

To induce TCF25 transcript formation *in vitro* by nonlinear splicing, circTCF25 over-expression vector was constructed. The specially designed front and back circular frames were synthesized and added to pCDH-CMV-MCS-EF1-copGFP for the circulation of transcripts. As previously reported^15^, the front circular frame contains the endogenous flanking genomic sequence with EcoRI restriction enzyme site, and the back circular frame contains part of the inverted upstream sequence with BamHI site. The cDNA encoding circTCF25 in Hela cells was amplified using primers 5′-CGGAATTCTGAAATATGCTATCTTACAGAGAGAGCGCTGTACAGCATGGA-3′ and 5′-CGGGATCCTCAAGAAAAAATATATTCACCTCCAGGGAACATGGTGAGCGC-3′. As a result, the 453bp target fragment orderly contains EcoRI site, splice acceptor AG, circTCF25 sequence, splice donor GT and BamHI site. Then, the amplified fragment was cloned to the vector between the two frames. Besides, we also established the mock vector only containing a nonsense stuffer between the two circular frames without the circTCF25 encoding cDNA. The result of vector construction was verified by direct sequencing. The vectors were constructed with the help of Guangzhou Geenseed Biotech Co, Guangzhou, China.

### Cell culture and gene transfection

All the cells were cultured in RPMI1640 medium (Gibco-BRL, Carlsbad, CA) with 10% fetal calf serum. Culture plates were incubated at 37 °C in a humidified atmosphere with 5% CO_2_. Cells were transfected with corresponding plasmids using the Lipofectamine 2000 (Invitrogen, Carlsbad, CA) in the light of the manufacturer’s recommendations. And the cells were harvested at 48 hour after transfection.

### Western blot

Done as described previously^37^, a total of 25 μg cellular protein was loaded onto 12% SDS PAGE, and then was transferred to PVDF membranes. After blocking for 2 hours, membranes were incubated with primary antibody of CDK6 (1:500 dilution, Santa Cruz, CA) at 4 °C overnight, followed by appropriate HRP conjugated secondary antibody under room temperature for 2 hours and ECL detection.

### Proliferation and migration assay

Wound healing and Edu assays were performed to evaluate the ability of proliferation and migration, as previously described^37^,^38^. The 5′-Ethynyl-2′-deoxyuridine Edu assay was done using the Cell-Light™ Edu DNA Cell Proliferation Kit (Guangzhou Ribobio Co., Guangzhou, China) according to protocols.

### Immunohistochemistry staining

The 5 μm sections were prepared from bladder cancer patients, and paired carcinoma and para-carcinoma tissues were analyzed. Immunohistochemistry staining was preformed as previously described^37^. Tissue sections were incubated overnight in primary antibody of CDK6 (1:200 dilution, Santa Cruz, CA), followed by bio-anti-rabbit secondary antibody for an hour. The intensity of staining was graded by a modified scoring method, immunostaining were scored by multiplying the intensity score (0 = negative, 1 = low, 2 = high) and the percentage of the total cell population (0 = none stained, 1 = 1~49% stained, 2 = 50~100% stained). The multiplied score less than one was taken for negative staining (−), one and two were identified as moderate staining (+), and four was considered to be intense staining (++).

### Statistical analysis

The Wilcoxon signed-rank test was performed to analyze significant differences regarding the expression level of circRNAs and miRNAs between samples. Pearson’s Coefficient was applied to compare the microarray data and qPCR results. Student’s t test (two-tailed) was performed for other data analysis. P < 0.05 was considered to be significant.

## Additional Information

**How to cite this article**: Zhong, Z. *et al*. Screening differential circular RNA expression profiles reveals the regulatory role of circTCF25-miR-103a-3p/miR-107-CDK6 pathway in bladder carcinoma. *Sci. Rep*. **6**, 30919; doi: 10.1038/srep30919 (2016).

## Supplementary Material

Supplementary Information

Supplementary Table S1

Supplementary Table S2

Supplementary Table S3

## Figures and Tables

**Table 1:** The top 20 up-regulated and down-regulated circRNAs ranked by fold changes in microarray data.

CircRNA ID	CircRNA type	Chrom	P-value	FDR	Fold Change	CircRNA ID	CircRNA type	Chrom	P-value	FDR	Fold Change
Up-regulated circRNAs						Down-regulated circRNAs					
hsa_circ_0000144	antisense	chr1	5.07786E-05	0.003413506	53.6447466	hsa_circ_0003266	exonic	chr3	0.000332588	0.006289773	17.3348448
hsa_circ_0000658	intronic	chr15	0.000109361	0.004059999	49.9942392	hsa_circ_0092342	intronic	chr11	0.001783464	0.012188042	14.2295363
hsa_circ_0023642	exonic	chr11	7.83405E-06	0.002278329	40.5013601	hsa_circ_0007158	exonic	chr5	0.000618958	0.007529281	10.4740421
hsa_circ_0002623	exonic	chr1	0.00016776	0.004908056	39.3568643	hsa_circ_0028173	exonic	chr12	0.000357005	0.006299116	10.3685193
hsa_circ_0032821	exonic	chr14	0.000179758	0.005083964	33.7353599	hsa_circ_0009361	exonic	chr1	0.006950979	0.024428337	9.2280246
hsa_circ_0008035	exonic	chr8	0.00031574	0.006174769	31.7607792	hsa_circ_0040039	exonic	chr16	0.005159873	0.020348207	8.2101043
hsa_circ_0005273	exonic	chr8	0.000955005	0.008931792	30.8923414	hsa_circ_0092340	intronic	chr1	1.53087E-05	0.002278329	7.1052824
hsa_circ_0084171	exonic	chr8	3.27139E-05	0.003199423	30.7866003	hsa_circ_0082582	exonic	chr7	1.22246E-05	0.002278329	6.3227161
hsa_circ_0058058	exonic	chr2	0.000379055	0.006446181	29.2960513	hsa_circ_0050867	exonic	chr19	1.04921E-06	0.001197149	6.0107321
hsa_circ_0061265	exonic	chr21	0.000200915	0.005083964	26.5662267	hsa_circ_0079480	exonic	chr7	0.001861507	0.01239677	5.9734844
hsa_circ_0001336	intronic	chr3	0.00953037	0.029284072	21.8702349	hsa_circ_0012185	exonic	chr1	0.000692426	0.007848263	5.8796393
hsa_circ_0041103	exonic	chr16	0.000650387	0.00759821	21.3622728	hsa_circ_0002435	exonic	chr10	5.64461E-06	0.002146833	5.7331469
hsa_circ_0047322	exonic	chr18	3.14432E-05	0.003165855	20.1999654	hsa_circ_0077526	exonic	chr6	0.002731199	0.014935657	5.6016692
hsa_circ_0072088	exonic	chr5	0.003426956	0.016380189	15.5158638	hsa_circ_0079440	exonic	chr7	0.001362999	0.010772358	5.6015307
hsa_circ_0008558	exonic	chr16	2.39297E-05	0.003165855	14.3819764	hsa_circ_0004354	exonic	chr16	0.001451444	0.01103342	5.5268668
hsa_circ_0041151	exonic	chr17	8.85135E-06	0.002278329	12.6338505	hsa_circ_0012634	exonic	chr1	5.61052E-05	0.003413506	5.4826496
hsa_circ_0011385	exonic	chr1	0.000475062	0.006832506	12.0846414	hsa_circ_0005265	exonic	chr20	0.000919338	0.008731033	5.2478181
hsa_circ_0060219	exonic	chr20	0.000516006	0.007122127	11.1073618	hsa_circ_0047585	exonic	chr18	0.002544392	0.014539989	5.0275224
hsa_circ_0000520	intragenic	chr14	5.59795E-06	0.002146833	10.4753353	hsa_circ_0031027	exonic	chr13	0.017406651	0.042468259	4.779503
hsa_circ_0003528	exonic	chr5	0.001072132	0.00950753	10.4486611	hsa_circ_0052877	exonic	chr2	7.18003E-05	0.003413506	4.4278205

Note: CircRNA ID: The circRNA ID is in circBase (http://circbase.mdc-berlin.de). CircRNA type: The circRNAs are classified into 5 types: “exonic”, “intronic”, “antisense”, “intragenic” and “intergenic”. P-value: P-value calculated from paired t-test. FDR: FDR is calculated from Benjamini Hochberg FDR. Fold Change: The absolute ratio (no log scale) of normalized intensities between two conditions.
