# Supplementary material for: Screening differential circular RNA expression profiles reveals the regulatory role of circTCF25-miR-103a-3p/miR-107-CDK6 pathway in bladder carcinoma
Source: Sci Rep. 2016 Aug 3;6:30919. doi: 10.1038/srep30919 (PMC4971518; doi:10.1038/srep30919)
Supplement: Supplementary Information [file srep30919-s1.pdf]

Screening differential circular RNA expression profiles reveals the regulatory role of circTCF25-miR-103a-3p/miR-107-CDK6 pathway in bladder carcinoma

Zhenyu Zhong, Mengxin Lv & Junxia Chen

Supplementary Figures

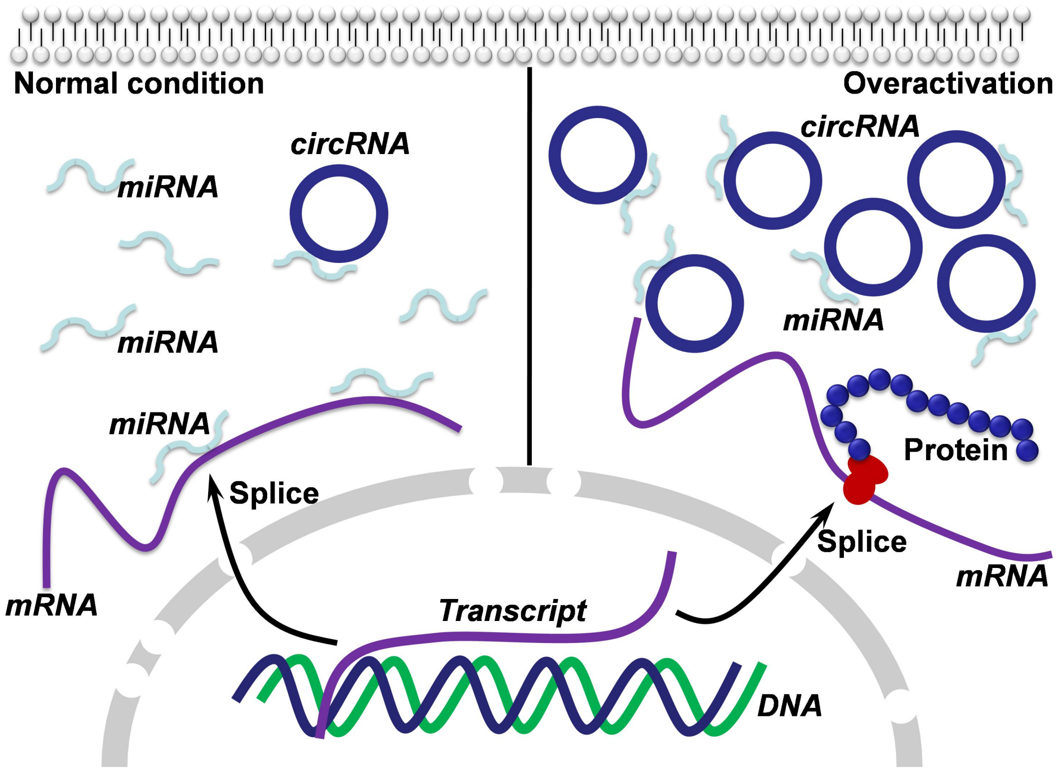

Supplementary Figure S1. Schematic strategy for circRNAs on post-transcriprional regulation. In normal condition, miRNAs play inhibiting effects by targeting mRNA 3' UTR. In comparison, with the circRNAs serving as target decoys, up-regulation of exogenous circRNAs enables them to sequester miRNAs and suppress their activity and function by binding on shared miRNA response elements (MREs). Free from miRNA-mediated inhibiting, the products of the target mRNAs increase.

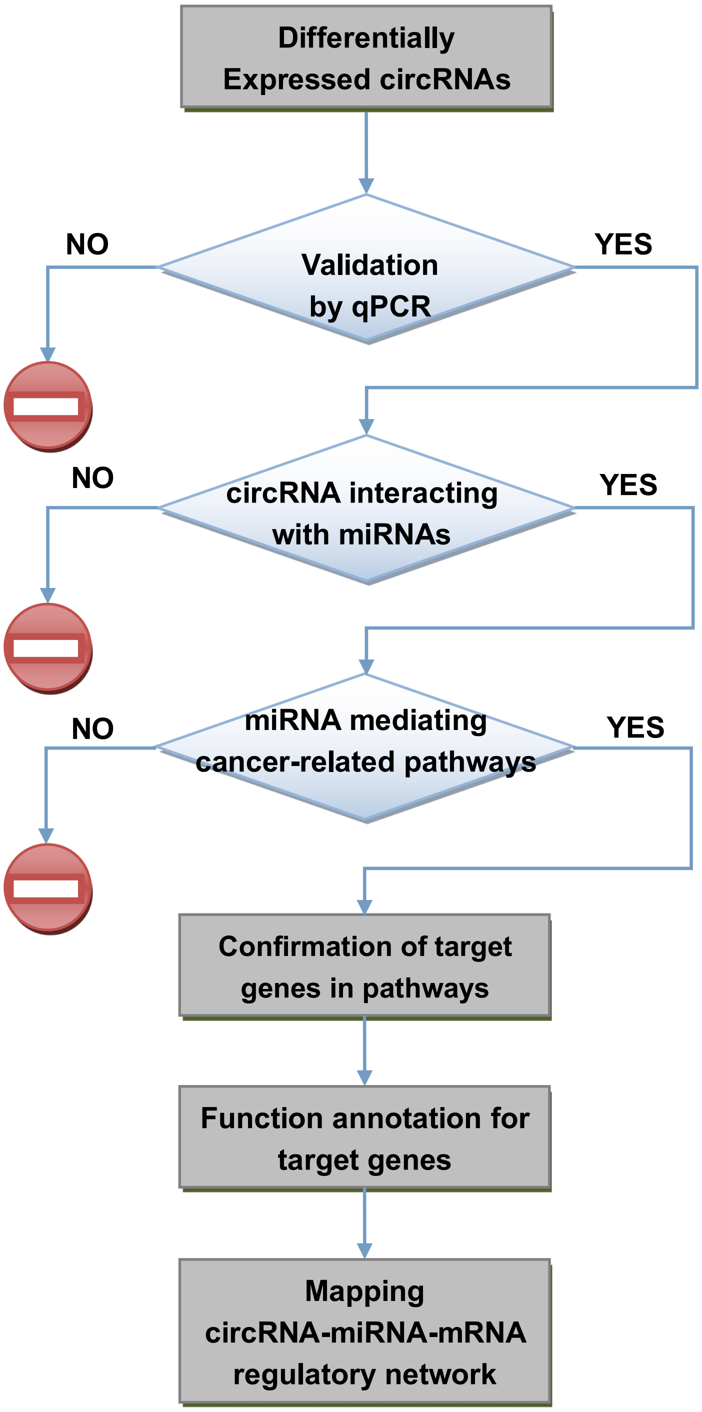

Supplementary Figure S2. Workflow depicting screening and analysis of cancer-related pathways in circRNA profiles with bioinformatical methods.

Supplementary Methods

Primers used in qRT-PCR. Sequences are written in 5'-3' direction.

RT-PCR:

circRNA and mRNA: Random primers

miR-103a-3p: GTCGTATCCAGTGCGTGTCGTGGAGTCGGCAATTGCACTGGATACGACTCATAGCC

miR-107: GTCGTATCCAGTGCGTGTCGTGGAGTCGGCAATTGCACTGGATACGACTGATAG

U6: CGCTTCACGAATTTGCGTGTCAT

qPCR:

circFAM169A forward: GAGGTAAAGATTTTGGGCTTCACA

circFAM169A reverse: GGATTTTCAGGGTCCCCACA

circTRIM24 forward: GGATATGATGGAAAGGCTTTTG

circTRIM24 reverse: AAACACTGGTCGCTGGCTG

circTCF25 forward: GATACAGCAGGCGCTCACCAT

circTCF25 reverse: TCGGGTCTGCGGTAATCCA

circZFR forward: GATTATCATACGCATTCTTCG

circZFR reverse: TTTCTGAACTGCCTGTAACTC

circPTK2 forward:TATTGGACCTGCGAGGGATT

circPTK2 reverse: TGTGAACCAGGGTAGCCAGAA

circBC048201 forward: CACCGTTCCTCCACTGTTCG

circBC048201 reverse: CCGGGTCCACTAGATGTCTGC

β-actin forward: CCTGTACGCCAACACAGTGC

β-actin reverse: ATACTCCTGCTTGCTGATCC

miR-103a-3p forward: GGGAGCAGCATTGTACAGGG

miR-103a-3p reverse: CAGTGCGTGTCGTGGAGT

miR-107 forward: GGAGCAGCATTGTACAGG

miR-107 reverse: CAGTGCGTGTCGTGGA

U6 forward: GCTTCGGCAGCACATATACTAAAAT

U6 reverse: CGCTTCACGAATTTGCGTGTCAT
